# Supplementary material for: Replication-dependent size reduction precedes differentiation in Chlamydia trachomatis
Source: Nat Commun. 2018 Jan 3;9:45. doi: 10.1038/s41467-017-02432-0 (PMC5752669; doi:10.1038/s41467-017-02432-0)
Supplement: Supplementary file 1 — Supplementary Information [file 41467_2017_2432_MOESM1_ESM.pdf]

Supplementary Figure 1. Novel three-dimensional electron microscopy approach for the comprehensive analysis of the chlamydial inclusion.

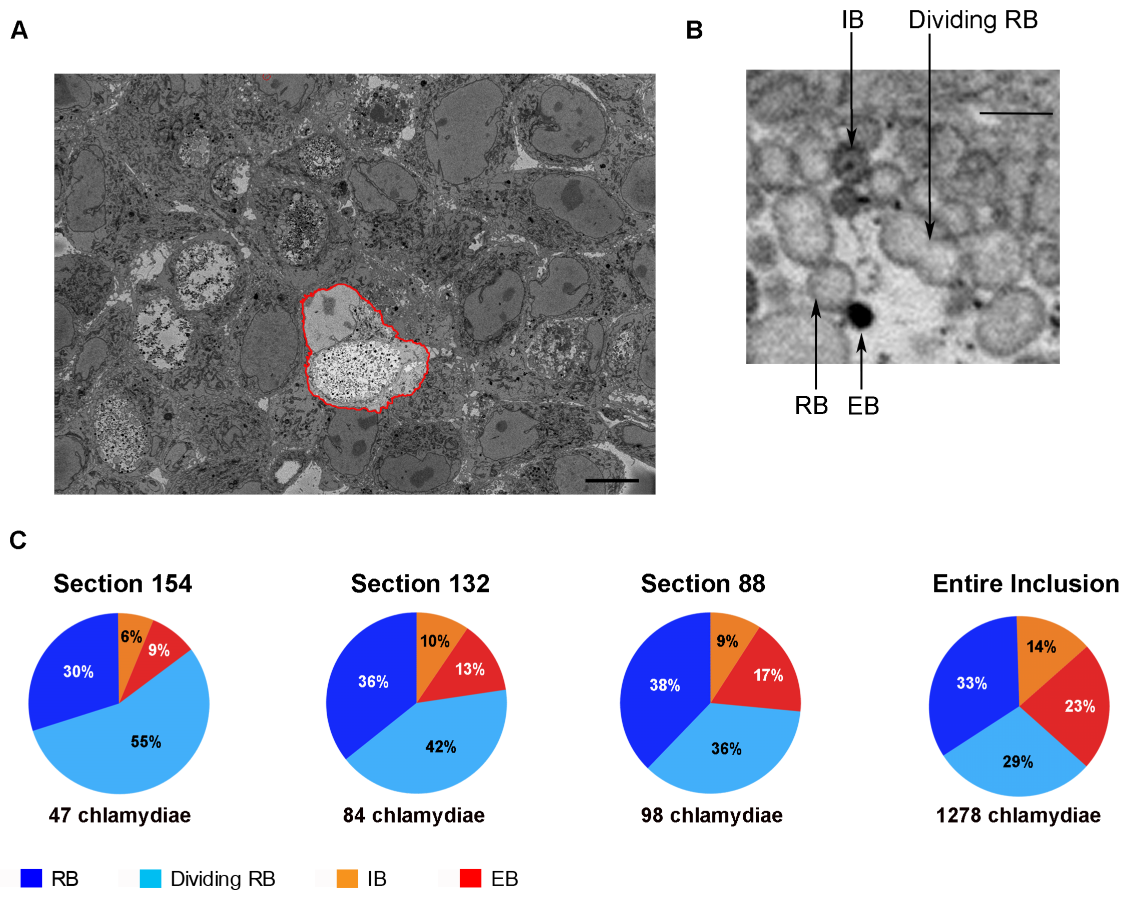

Supplementary Figure 1. Three-dimensional electron microscopy approach for the comprehensive analysis of the chlamydial inclusion.

(A) Representative monolayer of *C. trachomatis*-infected HeLa cells at 32 hpi. A single infected cell is outlined in red with the large chlamydial inclusion below the gray nucleus. Scale bar: 200 µm. **(B)** Magnified image of a single section showing four chlamydial developmental forms: reticulate body (RB), dividing RB, intermediate body (IB) and elementary body (EB). Scale bar: 1000 nm. **(C)** Pie charts showing the distribution of the four chlamydial forms in the entire inclusion and each of the three sections from Fig. 1A.

Supplementary Figure 2. Validation of chlamydial quantification.

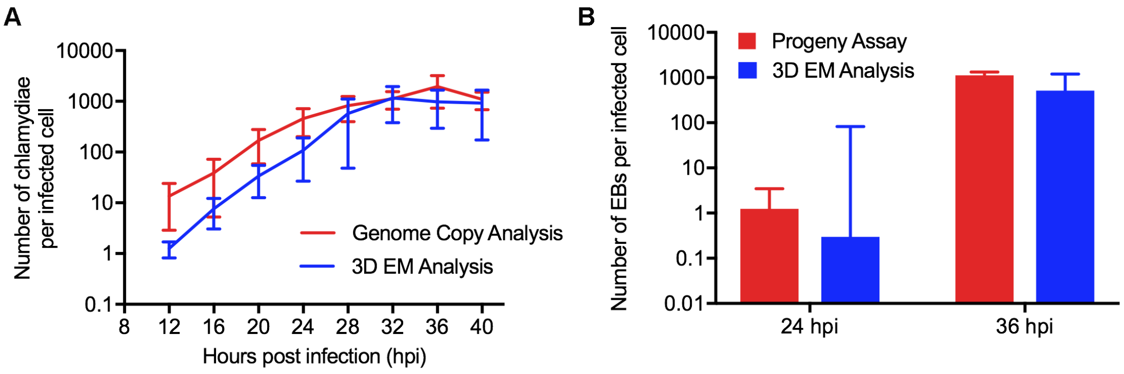

Supplementary Figure 2. Validation of chlamydial quantification.

(A) Total number of chlamydiae per infected cell determined by 3D EM analysis of *Chlamydia*-infected cells (12 hpi n=50 inclusions, 16 hpi n=31, 20 hpi n=22, 24 hpi n=10, 28 hpi n=13, 32 hpi n=10, 36 hpi n=9, 40 hpi n=10) and by measuring the number of genome copies by qPCR (n=3 independent experiments) [1]. Error bars indicate standard deviation from the mean. **(B)** Number of EBs per infected cell at 24 hpi (n=10 inclusions) and 36 hpi (n=9 inclusions), as measured by 3D EM analysis, and infectious EBs per cell measured with an infectious progeny assay (n=3 independent experiments) [2]. Error bars indicate standard deviation.

Supplementary Figure 3. Temporal analysis of IB and EB size.

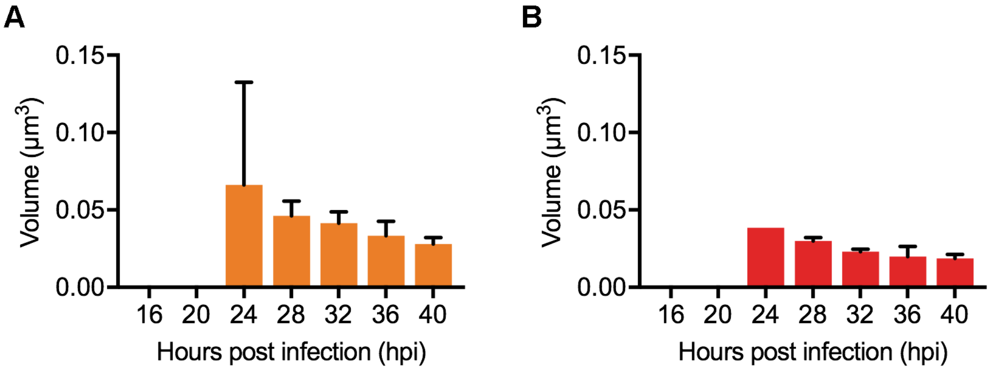

Supplementary Figure 3. Temporal analysis of IB and EB size.

(A) Measurements of IB volume and **(B)** EB volume during the developmental cycle. Average volume of all IBs and EBs in each inclusion was first determined, and then the mean volume for all inclusions at each time point was calculated. 24 hpi (n=3 inclusions), 28 hpi (n=5), 32 hpi (n=8), 36 hpi (n=5), 40 hpi (n=8). Error bars indicate standard deviation. No IBs or EBs were detected at 12, 16, or 20 hpi.

Supplementary Table 1. SBEM Micrograph Image Sizes

Time point (hpi)	Monolayer microscope ID	Pixel size (nm)
12	5221079	8.6 x 8.6 x 60
16	455561	32 x 32 x 60
20	5239437	10.4 x 10.4 x 60
24	5239072	26 x 26 x 60
28	5194144, 5194220, 5194245, 5194413	3.6 x 3.6 x.60
	5221161	13 x 13 x 60
	5230011, 5228936	10.3 x 10.3 x 60
32	5204491, 5206195	8.4 x 8.4 x 60
	5229784	10.3 x 10.3 x 60
	5239356	20 x 20 x 60
36	5119341, 5119366	4.6 x 4.6 x 60
	5120258	2.5 x 2.5 x 60
	5204439	8.4 x 8.4 x 60
	5204464	3.3 x 3.3 x 60
	5221104, 5228909	10.3 x 10.3 x 60
40	5203366, 5203391	8.4 x 8.4 x 60
	5229169	10.3 x 10.3 x 60

Supplementary Table 2. Selection of chlamydial inclusions for analysis by 3D EM

Inclusions marked in gray were randomly selected for segmentation and analysis.

20 HPI

Size Bin	Inclusion volume (μm^3)
1	**3.81**
	6.88
	9.36
	**18.32**
	19.27
	26.91
	28.06
	**28.55**
	**28.62**
	**32.9**
	**34.47**
	**34.54**
	**35.75**
2	35.98
	41
	**41**
	**48.85**
	**49.81**
	51.15
	**53.47**
	59.74
	**62.6**
	66.64
	**70.14**
	**72.11**
	73.51
3	**73.9**
	**74.7**
	76.09
	**76.1**
	**86.15**
	**95.02**
	105.64
	**107.81**
	114.65
	126.77
	158.54
	**189.46**
	226.18

24 HPI

Size Bin	Inclusion volume (μm^3)
1	**76.86**
	83.12
	93.99
	95.01
	107.38
	107.58
	**111.96**
	120.55
	131.54
	**132.4**
	133.25
2	**145.06**
	146.36
	**147.5**
	155.88
	158.49
	161.4
	**163.6**
	170.22
	183.27
	190.9
	191.73
3	**193.84**
	196.37
	**203.38**
	207.15
	268.24
	**270.47**
	280.11
	298.2
	317.06
	329.19

28 HPI

Size Bin	Inclusion volume (μm^3)
1	16.44
	49.14
	**63**
	190.35
	225.48
	228.51
	**239.7**
	**257.6**
2	**287.62**
	**288.23**
	**294.3**
	314.89
	**322.53**
	**337.96**
	341.77
	**380.2**
3	423.89
	453.34
	**754.79**
	**791.05**
	**819.9**
	**1474.33**
	1611.81

32 HPI

Size Bin	Inclusion volume (μm^3)
1	50.85
	151.14
	160.79
	**190.12**
	195.45
	262.62
	274.86
	282.7
	288.56
	**296.58**
	**322.48**
	352.48
	367.12
	369.19
	399.27
2	**412.72**
	418.2
	424.87
	447.88
	470.01
	475.89
	514.99
	557.72
	584.3
	590.53
	**591.15**
	**604.14**
	616.7
	660.31
	697.69
	**754.25**
3	784.17
	**796.53**
	865.65
	935.59
	1048.49
	1058.39
	1059.45
	1127.23
	**1246.11**
	1289.47
	1297.27
	1342.45
	1564.29
	1626.86

36 HPI

Size Bin	Inclusion volume (μm^3)
1	67.5
	81.03
	104.86
	110.92
	131.99
	151.06
	202.82
	**200.72**
	243.26
	244.98
	253.34
	299.65
	299.95
	313.82
	321.29
	345.89
	363.61
	**375.11**
	378.12
	382.24
	386.6
	**397.97**
2	430.85
	453.48
	461.36
	462.92
	464.02
	481.61
	488.49
	504.31
	510.82
	547.41
	**550.64**
	553.33
	555.24
	556.07
	**562.86**
	589.94
	601.66
	611.21
	615.6
	**660.1**
	669.08
	682.43
	693.27

40 HPI

Size Bin	Inclusion volume (μm^3)
1	8.75
	210.15
	222.58
	222.92
	**267.9**
	299.21
	**400.17**
	418.89
	504.76
	**504.99**
2	**564.25**
	617.86
	802.4
	**804.15**
	853.29
	1012.23
	1073.28
	1124.51
	**1269.42**
	**1300.33**
3	1411.72
	1452.91
	**1508.54**
	1554.42
	1663.13
	**1712.83**
	2023.79
	**2087.46**
	2861.2
	3767.52

	1682.98		763.7
	1737.26		763.99
			868.64
			912.95
			975.63
			1005.52
			1032.44
			1069.6
			1078.86
			1105.98
			1141.24
			1145.76
			1157.24
		3	1175.98
			1177.25
			1180.03
			1202.05
			1239.13
			1312.94
			1357.92
			1558.06
			1648.5
			1694.19
			1718.93
			1908.49

SUPPLEMENTARY NOTES

Supplementary Note 1: Introduction

In this appendix we carry out a detailed description of a mathematical model used in the main text to provide insight into the experimental data and the proposed mechanisms for *Chlamydia* cell fate regulation. The experimental evidence presented in the main text points to the intriguing hypothesis that cell size could regulate conversion. As RB cells divide, they become increasingly smaller on average, and a threshold size would initiate EB conversion with high probability.

In order to accurately reflect the diversity of RB sizes in an inclusion at any given time, as well as the randomness inherent in RB growth rates and division, we developed a stochastic model of a chlamydial infection. Individual RBs were allowed to grow at slightly different rates from each other and divide at different time intervals, resulting in a broad distribution of RB sizes. We also include in the model the fact that Chlamydia cell division is slightly asymmetric, which also increases the size variability over time.

When RBs reach a threshold size, they have a given probability per unit time of initiating conversion. Since it takes several cell divisions to reach the threshold size, this mechanism implements a delay before conversion and thereby the conversion strategy outlined above.

This model is consistent with the experimental data both of the populations over time of each of the four chlamydial forms, and also regarding their size. This model provides evidence that size control is a viable strategy to optimize the number of infectious bacteria during an infection, which is consistent with experimental measurements in *Chlamydia*.

Supplementary Note 2: Model Description

The stochastic model used in Figure 5 of the main text has the aim to show how a cell size regulatory mechanism can effectively implement a strategy of early replication followed by late RB-to-EB conversion. A simulation of this model begins with a single RB inside a cytoplasmic inclusion growing into a dividing RB (also known as DB). The DB eventually divides into two RBs, each of which in turn grows and divides, and so on, modeling the growth of the chlamydial population within a single inclusion. At every time point, each RB and DB present has an assigned size. When this size decreases below a threshold value s_{thr}, the RB has the ability to convert into an IB and subsequently an EB. See Figure 4B of the main text for a network of the transitions between chlamydial forms.

The time between state transitions is determined stochastically by drawing numbers independently from a continuous distribution. Rather than an exponential distribution as is common for chemical reactions, we use a gamma distribution, which represents the time before a given number of events in a Poisson processes, and which better describes the timing between cell transitions. For instance, the time for the transition from RB to DB has a gamma distribution with mean ρ_{RD}, where ρ_{RD} is the average time before the transition. Similarly for transitions from DB to RB, from RB to IB, and from IB to EB. The shape parameter for all gamma distributions used is $\gamma_s = 5$. Since RBs can transform into either

DB or IB, separate times are drawn for each outcome from gamma distributions, and the earlier of the two times determines the actual cell fate.

Gamma distributions are a natural choice for the time between divisions, because they explicitly postulate a number of events that need to take place before division can occur, with each of these events assuming an exponential distribution for simplicity. They have been used before for this purpose, see for instance [3, 4]. An exponential distribution itself would be considered too noisy to represent time between divisions. Other distributions are also possible, for instance a lognormal distribution. In fact gamma and lognormal distributions can look very similar, and it may be quite difficult to distinguish between them experimentally – see for example Figure 1 in [3].

The mean transition time ρ_{RI} is a function of the current size s, namely it is set to ∞ if $s \geq s_{thr}$. This effectively shuts down conversion above the threshold size and implements the size control described above. Since this model describes the dynamics of different chlamydial forms inside an inclusion, we have also implemented a form of population capacity for this system. When a maximum of 1000 chlamydiae are present, further cell division is inhibited by preventing additional RB-to-DB transitions.

Regarding the cell size dynamics, each time that an RB is created a growth rate k is calculated from a normal distribution with mean μ_R and standard deviation σ_R. If Δt is the time before the next state transition, then the size at the new state transition is $e^{k\Delta t}$ times the size at the previous transition. The same calculation is carried out for every DB, using a new independent growth rate k sampled using the same parameters μ_R, σ_R. At the end of each DB state, the model implements asymmetric cell division as follows. The cell is partitioned in two using a ratio r sampled from a binomial partition distribution distribution, $r \sim \frac{1}{n}Binom(n, 1/2)$. This distribution has mean $E(r) = 1/2$ and standard deviation $\sigma_1 = \frac{1}{2\sqrt{n}}$. One way to conceptualize it is to divide the DB into n different compartments, each of which chooses one of the two daughter cells independently using a fair coin.

Binomial distributions are also used in the literature as a simple partitioning method for cell division, see for instance [5, 6]. Once again they are not the only proposed method, but other methods tend to be compared with binomial partitioning. There is an inherent appeal in this distribution because of a simple (if not entirely realistic) mechanistic assumption that can lead to it, that of different components of the cell choosing daughter cells independently.

A simple calculation writing $r = \frac{1}{n}\sum_{j=1}^{n} X_j$, where X_j is a Bernoulli variable, shows that the product of two independent binomial partition functions is also a binomial partition, $r_1 r_2 \sim \frac{1}{n^2}Binom(n^2, 1/4)$. More generally,

$$r_1 \dots r_m \sim \frac{1}{n^m}Binom(n^m, \frac{1}{2^m}).$$

This product has mean $E = \frac{1}{2^m}$, variance $\sigma^2 = \frac{1}{n^m}2^m(1-2^m) \approx \frac{1}{n^m}2^m = (2/n)^m$, and coefficient of variation

$$CV = \sigma/E \approx (2/n)^{m/2}\, 2^m = (2/n)^{m/2}\, 4^{m/2} = (8/n)^{m/2}.$$

If the value chosen for n is significantly larger than 8, $n \gg 8$, then the coefficient of variation becomes very small for large m, so that $r_1 \dots r_m \approx \frac{1}{2^m}$.

Suppose that $t_1, t_2, \ldots 2m$ are the times of the state transitions with $\Delta_i = t_i - t_{i-1}$, and s_i is the size at time t_i. Assuming that the size threshold for conversion has not been reached, it holds for an RB-to-DB transition that

$$s_{i+1} = s_i e^{k_i \Delta_i}, \quad k_i \sim N(\mu_R, \sigma_R^2),$$

and for a DB-to-RB transition that

$$s_{i+1} = s_i r_i e^{k_i \Delta_i}, \quad k_i \sim N(\mu_R, \sigma_R^2), r_i \sim \frac{1}{n} Binom(n, 1/2)$$

Overall, these two transitions will alternate and it holds

$$s_{2m} = s_0 r_2 r_4 \ldots r_{2m} e^{k_1 \Delta_1 + \ldots + k_{2m} \Delta_{2m}}, \quad k_i \sim N(\mu_R, \sigma_R^2), \quad r_{2i} \sim \frac{1}{n} Binom(n, 1/2)$$

For $n \gg 8$, we have calculated above that $r_2 r_4 \ldots r_{2m} \approx 1/2^m$. The distribution of s_{2m} is roughly lognormal due to the Central Limit Theorem. This can also be roughly observed in the experimental data, see Figure 5B and 5C showing the size distribution of RBs at 24hpi and 40hpi. The conversion of RB to IB and EB acts as a 'drain' on this distribution as chlamydiae become sufficiently small. Notice also that a lognormal distribution is independent of the choices for the distribution of k_i and Δ_i, so by itself it does not validate these choices.

If an RB undergoes a single transition to DB and cell division to form a new RB, the size ratio for the new RB is

$$R = s_{i+2}/s_i = r e^{k_i \Delta_i} e^{k_{i+1} \Delta_{i+1}}.$$

The mean and standard deviation for this size ratio can be calculated as a function of the parameters of the system, and for the chosen parameters it holds that $E(R) \approx 0.796$, $\sigma(R) \approx 0.156$. Thus a DB divides on average when it reaches about 1.6 ± 0.31 times the size of the original RB.

Supplementary Note 3: Stochastic Model Parameters

The parameter values used for this simulation are the following. The simulation is started at time $t_0 = 12$ hpi, and the maximum computed time is 40 hpi, as measured experimentally. The initial RB size is $s_0 = 1\mu\ell^3$, as measured in Figure 3A. The threshold below which RBs can convert is set to $s_{thr} = 0.06\mu\ell^3$, as measured in Figure S3A.

The mean transition time ρ_{RD} is used to draw transition times from a gamma distribution with shape parameter $\gamma_s = 5$ and scale parameter $\theta = \rho_{RD}/\gamma_s$. Similarly for all other transitions. In order to measure mean transition times between RB and DB, notice in Figure 1B that inclusions grew from a mean of 1.3 chlamydiae at 12hpi to a mean of 577 chlamydiae at 28hpi. This is a $440 = 2^{8.8}$-fold growth, leading to an estimate of around 8.8 cell divisions over a span of 16 hours. This leads to an estimate of around 1.8 hours per cell division. Since also in Figure 1B one can see that the numbers of RB and DB are roughly similar at all times, we estimate that half of that time, or 0.9 hours, is spent on average in each of RB and DB forms. In this way we set the mean transition times $\rho_{RD} = \rho_{DR} = 0.9h$. We also set $\rho_{IE} = 2.5h$ and $\rho_{RI}(s) = 0.1h$ when $s \leq s_{thr}$, $\rho_{RI}(s) = \infty$ otherwise (i.e. conversion is shut down). These parameters determine the size of the IB and EB populations and were set for consistency with experimental data in Figure 5.

Regarding the growth rates for chlamydial forms, notice that on Figure 3B there is a remarkable stability in the ratio of mean DB to RB volume ratio of around 1.5. The ratio of DB to daughter RB can be described using the above variables as

$$s_i/s_{i+1} = s_i/(s_i r_i e^{k_i \Delta_i}) = 1/(r_i e^{k_i \Delta_i}).$$

Setting the growth rate $\mu_R = 0.25h^{-1}$, the above ratio has a mean of 1.57 as measured by simulation. This is consistent with the experimental data for this ratio. We also set the growth variability parameter $\sigma_R = 0.04h^{-1}$.

The asymmetric cell division parameter σ_1 has been directly measured experimentally using 3D microscopy techniques to have a value of around $\sigma_1 = 0.05$, as observed in Figure 4. Since the partitioning of the cell division is calculated through a binomial distribution $r \sim \frac{1}{n} Binom(n, 1/2)$ and $\sigma_1 = \frac{1}{2\sqrt{n}}$, this formula can be used to calculate the value of n, $n = 100$. Notice that $n \gg 8$, as required for part of the analysis above.

Supplementary Note 4: Size Structured Population Density

We conclude with a plausible analytical representation of the size-structured model in Figure 5. Suppose again that each Chlamydia in the system has an associated size, and that conversion to EB can only take place when the size has become sufficiently small. For a two-variable system involving the forms EB and RB, a simple size-structured model is given by the work by Diekmann and colleagues [7]. Let $R(x, t)$ represent the density of the population of RB forms with size x at time t. So for any two sizes $x_1 < x_2$, the number of RB forms at time t is given by $\int_{x_1}^{x_2} R(x, t)\, dx$. The dynamics of the system, assuming deterministic growth and symmetric cell division, is described by the equation

$$\frac{\partial R}{\partial t}(x, t) = -\frac{\partial}{\partial x}(kxR(x, t)) - \alpha_{RR} R(x, t) + 4\alpha_{RR} R(2x, t) - \mu(x) R(x, t).$$

Here kx is the linear growth rate of an RB of size x, and the first term on the right hand side describes the growth drift in the system. The second and third terms describe the rate of cell division – the density $R(x, t)$ is positively affected by the density $R(2x, t)$ since the cells with size $2x$ are dividing with rate α_{RR}. The function $\mu(x)$ is the rate of degradation, or in this case conversion to EB, depending on size. We define $\mu(x) = 0$ for $x > s_{thr}$ and $\mu(x) = \alpha_{RE} > 0$ for $x \leq s_{thr}$. Finally, the EB forms do not need to be size structured and can be defined by the equation

$$E'(t) = \int_0^\infty \mu(x) R(x, t)\, dx = \alpha_{RE} \int_0^{s_{thr}} R(x, t)\, dx.$$

This model was studied in detail in [7], showing that under certain conditions a stable size distribution is reached. However in our case we don't expect a stable equilibrium distribution, because the rate of cell division is significantly faster than cell growth.

A generalization to the four variable case can be written as follows, including density functions for $R(x, t), D(x, t)$ and functions $E(t), I(t)$. For simplicity we assume symmetric cell divisions.

$$
\begin{aligned}
\frac{\partial R}{\partial t}(x,t) &= -\frac{\partial}{\partial x}(kxR(x,t)) - \alpha_{RD}R(x,t) + 4\alpha_{DR}D(2x,t) - \mu(x)R(x,t) \\
\frac{\partial D}{\partial t}(x,t) &= -\frac{\partial}{\partial x}(kxD(x,t)) + \alpha_{RD}R(x,t) - \alpha_{DR}D(x,t) \\
I'(t) &= \alpha_{RI}\int_0^{s_{thr}} R(x,t)\,dx - \alpha_{IE}I \\
E'(t) &= \alpha_{IE}I.
\end{aligned}
$$

Stochasticity in the growth rates can be incorporated through diffusion terms, see e.g. [8]. The similarity between this model and the simulations in Figure 5 is tempered by the fact that the numerical data simulates a single inclusion, with significant correlation between cells of similar lineages. Also, it is not clear how the choice of a gamma distribution for the cell division time, rather than an exponential distribution, affects the relationship with this model. The analysis of this model, and a full derivation based on the stochastic definition in the previous section, is out of the scope of this appendix and will be left for a future publication.

References

[1] S. Mathews, K. Volp and P. Timms, Development of a quantitative gene expression assay for *Chlamydia trachomatis* identified temporal expression of σ factor. FEBS Letters 458:354-358, 1999

[2] W. Beatty, R. Morrison and G Byrne, Reactivation of persistent *Chlamydia trachomatis* infection in cell culture, Infection and Immunity 63:199-205, 1995

[3] D. Antunes and A. Singh, Computing mRNA and protein statistical moments for a renewal model of stochastic gene-expression. Conference on Decision and Control, doi 10.1109/CDC.2013.6761031, 2014

[4] C. Pin and J. Baranyi, Single-cell and population lag times as a function of cell age, Appl. and Environ. Microbiol. 74(8):2534-2536, doi:10.1128/AEM.02402-07, 2008.

[5] D. Huh and J. Paulsson, Random partitioning of molecules at cell division, Proc. Natl. Acad. Sci USA 108:36:15004-15009, 2011.

[6] J. Loyd-Price, H. Tran, A. Ribeiro, Dynamics of small genetic circuits subject to stochastic partitioning in cell division, Journal of Theoretical Biology 356:11-19, 2014.

[7] Diekmann, O., Heijmans, H.J.A.M, and Thieme, H.R., On the stability of the cell size distribution. Journal of Mathematical Biology 19:227-248, 1984.

[8] Perthame, B. Introduction to structured equations in biology. Lecture notes, available at https://www.math.cmu.edu/cna/LectureNotesFiles/Perthame.pdf
optimal control processes. Interscience Publishers, New York, 1962.
